# Determination of the minimum infusion rate of alfaxalone during its co-administration with midazolam in goats

**DOI:** 10.1136/vetreco-2014-000065

**Published:** 2015-02-25

**Authors:** T. B. Dzikiti, P. S. Ndawana, G. Zeiler, L. Bester, L. N. Dzikiti

**Affiliations:** 1 Department of Companion Animal Clinical Studies, University of Pretoria, Onderstepoort, Republic of South Africa; 2 Department of Clinical Veterinary Studies, University of Zimbabwe, Harare, Zimbabwe; 3 School of Health Systems and Public Health, University of Pretoria, Pretoria, Republic of South Africa

**Keywords:** Anaesthesia, alfaxalone, midazolam, Total intravenous anaesthesia (TIVA), Minimum infusion rate (MIR)

## Abstract

**Introduction:**

The minimum infusion rate (MIR) of alfaxalone when co-administered with midazolam in goats was evaluated.

**Materials and methods:**

Eight goats (four does and four wethers) were anaesthetised, on separate occasions, with alfaxalone at an initial dose of 9.6 mg/kg/hour combined with one of three midazolam treatments: a bolus of 0.1 mg/kg followed by constant rate infusion (CRI) of 0.1 mg/kg/hour (treatment LMID), 0.3 mg/kg followed by CRI of 0.3 mg/kg/hour (MMID), 0.9 mg/kg followed by CRI of 0.9 mg/kg/hour (HMID), intravenously. Responses to stimulation (clamping on the proximal part of one digit of the hoof with Vulsellum forceps for 60 seconds) were tested every 30 minutes. In the absence or presence of a response to stimulation, the infusion rate was reduced or increased by 1.9 mg/kg/hour. Alfaxalone MIR was calculated as the mean of the infusion rates that allowed and abolished movement. Cardiopulmonary parameters were measured.

**Results:**

Alfaxalone MIR was 6.7 (6.7–8.6) mg/kg/hour, 6.7 (4.8–6.7) mg/kg/hour and 2.9 (1.0–4.8) mg/kg/hour for LMID, MMID and HMID respectively. Cardiopulmonary function was minimally affected, with hypoxaemia observed two minutes into anaesthesia during all treatments. Recovery from anaesthesia was excitement-free.

**Conclusions:**

Midazolam causes a dose-dependent reduction of alfaxalone MIR in goats. Oxygen supplementation is recommended during anaesthesia with alfaxalone and midazolam in goats.

## Introduction

Research on total intravenous anaesthesia (TIVA) in goats has increasingly become popular over the past few years (Ghurashi and others 2009, Dzikiti and others 2010, Dzikiti 2013, Ndawana and others 2014). General anaesthetic drugs such as propofol or alfaxalone are usually the foundation to which other ancillary anaesthetic drugs such as analgesics, sedatives, or muscle relaxants can be conjoined to complete TIVA protocols (Dzikiti 2013). Benzodiazepines such as midazolam contribute to TIVA through sedation and muscle-relaxation. Investigation of the anaesthetic-sparing effects of midazolam in alfaxalone-based TIVA may contribute new knowledge to the existent body of information on TIVA in goats.

Midazolam (8-chloro-6-(2-fluorophenyl)-1-methyl-4H-imidazol-(1,5-α)(1,4)-benzodiazepine), reduces anaesthetic requirements of general anaesthetic agents in various animal species, including goats (Taira and others 2000, Hendrickx and others 2008, Dzikiti and others 2011). Midazolam is a water-soluble, fast-acting benzodiazepine that acts through potentiation of neural inhibition that is mediated by gamma amino butyric acid and is also a centrally acting muscle relaxant (Farkas and others 1989, Lemke 2007, Olkkola and Ahonen 2008). It is associated with minimal adverse cardiovascular effects when administered intravenously at doses of 0.1–0.5 mg/kg for preanaesthetic medication (Lemke 2007, Dzikiti and others 2009, Ghurashi and others 2009, Dzikiti and others 2014) and at doses of 0.1–0.9 mg/kg/hour for an adjunctive role to general anaesthetic drugs in goats (Dzikiti and others 2010, Dzikiti and others 2011).

Alfaxalone is a steroid-type general anaesthetic drug commonly administered intravenously that can be used for induction and maintenance of general anaesthesia in various species, including goats (Klöppel and Leece 2011, Andaluz and others 2012, Mathis and others 2012, Suarez and others 2012, Ndawana and others 2014). Alfaxalone's pharmacokinetic profile after intravenous administration is typified by a rapid onset of action, rapid redistribution and a short terminal half-life (Ferré and others 2006, Suarez and others 2012). Alfaxalone‘s anaesthetic induction dose and minimum infusion rate (MIR) for anaesthetic maintenance in un-sedated goats have recently been reported as 3 mg/kg (Dzikiti and others 2014) and 9.6 mg/kg/hour (Ndawana and others 2014), respectively. Premedication with midazolam at 0.3 mg/kg reduces the dose of alfaxalone required for induction of general anaesthesia in goats by 33 per cent (Dzikiti and others 2014).

MIR represents the 50 per cent effective dose (ED_50_) for preventing movement response to a standardised supra-maximal noxious stimulus during TIVA and would be analogous to minimum alveolar concentration (MAC) for inhalation anaesthetic agents (Sear and others 1983, Hall and Chambers 1987). Factors that can influence MIR are similar to those influencing MAC and include, pharmacokinetic properties of the drug, age, physical status and body temperature of the patient, as well as concurrent administration of other drugs (opioids, sedatives) and the anaesthetic responsiveness of the CNS (Quasha and others 1980, Kaul and Bharti 2002).

The feasibility of coadministration of alfaxalone with midazolam for TIVA in goats has not been previously reported. The aim of the present study was to determine the MIR of alfaxalone required to prevent purposeful movement of the extremities in response to clamping on the proximal (soft) part of one digit of the hoof a claw with Vulsellum forceps during its co-administration with different constant rate infusion (CRI) doses of midazolam in goats.

## Materials and methods

### Background information and preparation for the experimental procedure

The present study was approved by the relevant institutional animal ethics committee (Certificate number: V028/13). The research site for the study is situated at a height above sea level of 1252 m; thus normal barometric (atmospheric) pressure ranges from 651 to 668 mm Hg. Eight clinically healthy goats (four does and four wethers) were used. The goats were assigned to three treatments in a randomised cross-over pattern, with a four-week washout period between treatments, in which general anaesthesia was achieved by combining alfaxalone with low dose midazolam (LMID treatment), moderate dose midazolam (MMID treatment) or high dose midazolam (HMID treatment). The alfaxalone MIR in goats reported in an earlier study to be 9.6 mg/kg/hour was used as the initial infusion rate in the present study. The goats’ median (range) age was 21 (21–21) while weight was 28.0 (23.9–31.6) kg at the beginning of the present study. The health status was assessed by physical examination, complete blood count and serum biochemical analysis; all findings were normal. Food and water were withheld for 18–24 hours before anaesthesia.

The goats were weighed on an electronic scale 30 minutes before the beginning of the experimental procedure. Baseline heart rate (HR), respiratory rate (fR) and rectal temperature were measured, after which the goats were placed on a custom-made sling-cum-table to ease restraint. Indwelling catheters (arterial and venous) were inserted before administration of any anaesthetic drugs and were well tolerated by the goats. A 24 standard wire gauge (SWG) catheter (Jelco, Medex Medical, Rossendale, Great Britain) was inserted percutaneously into the central auricular artery to facilitate continuous measurement of arterial blood pressures [systolic (SAP), diastolic (DAP) and mean arterial pressure (MAP)] and intermittent collection of arterial blood samples for gas analyses. Into each cephalic vein, a 20 SWG catheter (Jelco, Medex Medical, Rossendale, Great Britain) was inserted for later administration of intravenous fluids and midazolam (right thoracic limb), and alfaxalone (left thoracic limb). Lactated Ringer's solution (Intramed Ringer-Lactate Fresenius, Intramed, Port Elizabeth, South Africa) was administered, starting from five minutes before the administration of the ancillary anaesthetic agent (midazolam) to the time of termination of administration of the anaesthetic drugs (midazolam and alfaxalone), at a rate of 4 ml/kg/hour intravenously using a volumetric fluid infusion pump (Infusomat Space, B. Braun Medical, Bethlehem, USA).

### Experimental procedure

Induction of general anaesthesia involved initial administration of a bolus dose of midazolam (Dormicum, Roche Products, Isando, South Africa) at 0.1, 0.3, or 0.9 mg/kg intravenously over a two-minute period using a volumetric syringe-driving pump (Perfusor Space; B. Braun Medical, Bethlehem, USA) for treatment LMID, MMID and HMID, respectively, followed by a single anaesthetic induction bolus dose of alfaxalone (Alfaxalone-CD RTU, Jurox Pty Ltd, Rutherford, Australia) at 2.0 mg/kg administered using a volumetric syringe-driving pump over a 30-second period. If needed, an incremental bolus of alfaxalone at 0.5 mg/kg was administered every 15 seconds until depth of anaesthesia was sufficient, as judged by the presence of a weak medial palpebral reflex and adequate relaxation of the jaws to allow intubation of the trachea. The treatment (midazolam) dose was calculated, drawn up and injected by a person other than the principle investigator (TBD) who was blinded for later determination of alfaxalone MIR and assessment of quality of induction and recovery score. With the aid of an illuminated laryngoscope, an endotracheal tube (silicone type with an internal diameter of 7.5 mm) was inserted into the trachea while the goats were restrained into sternal recumbency. The cuff of the endotracheal tube was inflated to prevent leakage of gases up to a pressure of 20 cm H_2_O within the breathing circuit. The goats were then placed in right lateral recumbency. Quality of anaesthetic induction was scored on 0–2 score scale with zero representing failed intubation and two representing an excitement-free and easy intubation (Table 1).

**TABLE 1:** Scoring system used for quality of induction of and recovery from anaesthesia in goats

Score	Induction	Recovery
0	Excitement, vocalises, jumps or attempts to stand after becoming recumbent, unable to place orotracheal tube	Rough (several uncoordinated attempts to stand and ataxic)
1	Mild signs of excitement, some struggling, may or may not be intubated within 60 seconds	Relatively rough (several coordinated attempts to stand and ataxic)
2	Excitement-free induction, no outward sign of excitement, tracheal intubation easy	Relatively calm (1–2 coordinated attempts to stand with minimal short-lived ataxia)
3	–	Excitement-free (1 successful attempt to stand)

The goats were allowed to breathe spontaneously without any oxygen supplementation during the first two minutes of general anaesthesia so that the impact of the induction regimens on respiratory function could be assessed. Two minutes after completion of induction of general anaesthesia, the endotracheal tube was connected to a circle breathing circuit (Anaesthesia Systems, Clinicare; Crest Health Technology, Chatham, UK) with oxygen flow rate at 0.5 l/minute. Assisted ventilation (mechanical) was planned as a rescue intervention measure if end-tidal carbon dioxide tension rose above 55 mm Hg or if peripheral haemoglobin oxygen saturation (SpO_2_) was below 90 per cent at any time thereafter.

Immediately after completion of administration of the last dose of alfaxalone for induction, alfaxalone intravenous infusion was initiated for maintenance of general anaesthesia at an initial dose of 9.6 mg/kg/hour using a volumetric syringe-driving pump. Concurrent with the alfaxalone infusion, midazolam CRI was begun also using a volumetric syringe-driving pump at doses of 0.1, 0.3, or 0.9 mg/kg/hour for treatments LMID, MMID and HMID, respectively. The initial infusion rate of alfaxalone was maintained for 30 minutes before testing for responses to the noxious stimulus. This procedure was always carried out by the same investigator (TBD).

Determination of the MIR of alfaxalone in response to midazolam treatments involved application of the noxious stimulus, which was Vulsellum forceps clamped on the proximal (soft) part of one digit of the hoof just below the coronary band (incorporating the soft proximal part of the wall of the hoof, the distal phalanx and the bulb of the hoof) for 60 seconds or until occurrence of purposeful movement of the extremities; followed by adjustment of alfaxalone infusion rate according to the response to stimulation. Purposeful movement was strictly defined as gross movement of the head or limbs. Twitching of the stimulated limb was not regarded as a positive response. Non-purposeful movements such as shivering, stiffening and respiratory pattern changes were ignored. Digit clamping was done in a clockwise manner around the goat's four digits on the two non-dependent (left thoracic and pelvic) limbs starting with the medial digit of the left thoracic limb. In the absence of purposeful movement, the alfaxalone infusion rate was reduced by 1.9 mg/kg/hour (a fifth of the starting infusion rate) and held constant for another 30 minutes before application of a subsequent noxious stimulus. This activity was repeated until a purposeful response occurred. In the event of observation of an initial positive response (purposeful movement), the alfaxalone infusion rate adjustments were performed in reverse order. The MIR of alfaxalone in response to the midazolam treatments was calculated as the arithmetic mean of the alfaxalone infusion rates that allowed and abolished purposeful movement.

Following determination of the MIR, the alfaxalone and midazolam infusions were discontinued. The endotracheal tube was disconnected from the circle breathing circuit, following which the goats were placed on a rubber surface and assisted into sternal recumbency (against a cushioned wall) for recovery from general anaesthesia. Once the swallowing reflex was regained, the endotracheal tube was removed while still partially inflated to ensure that any fluid or particulate matter that might have been trapped by the cuff was dislodged from the trachea. Times (minutes) between the termination of anaesthesia and extubation, standing and voluntary motion were recorded. The quality of recovery was scored on a four-point scale from 0 to 3 with zero representing the worst possible quality of recovery and three representing an excitement-free recovery (Table 1).

### Physiological parameters measurement

A multi-parameter monitor (Cardiocap/5, Datex-Ohmeda Corporation, Helsinki, Finland) was used to measure basic physiological parameters continuously throughout the period of general anaesthesia.

Three electrocardiography (ECG) electrodes were placed on shaven areas (on the middle of the left shoulder, on the midline 2 cm in front of the point of the sternum and on the midline 2 cm cranial to the tip of the xiphoid) to provide a lead II ECG tracing. Haemoglobin oxygen saturation (SpO_2_) was measured using a pulse-oximetry probe placed around the tongue. Arterial blood pressure readings were obtained directly through a catheter pre-inserted into the central auricular artery to which a recently calibrated electronic strain gauge transducer (DTX Plus transducer, BD Medical, Johannesburg, South Africa) linking to the multiparameter monitor was connected. The arterial blood pressure measuring apparatus had been recently calibrated against a mercury column. For transducer calibration to atmospheric pressure, the scapulo-humeral joint or the point of the sternum were used as zero reference points in sternally recumbent or laterally-recumbent goats, respectively. Inspired and expired concentrations of carbon dioxide and oxygen were measured using side-stream spirometry, with side-stream gas sampler placed between the endotracheal tube and the Y-piece of the breathing system. The gas analyser had been calibrated with calibration gas as recommended by the manufacturer recently and would automatically self-calibrate to atmospheric air at the beginning of the experiment. Temperature was measured using an oesophageal probe placed as close to the base of the heart as possible. The targeted oesophageal temperature range was 37.0°C and 39.5°C. The targeted temperature was attained using a forced warmed air blanket (Bair Hugger; Augustine Medical, Minnesota, USA) and ordinary blankets placed around the goats.

The physiological parameters including HR, arterial blood pressure (SAP, DAP, MAP), body temperature and respiratory were measured continuously during the anaesthetic period, but were recorded, initially, before induction of general anaesthesia (baseline value), at 2 and 10 minutes after induction of anaesthesia and at 10-minute intervals thereafter.

Arterial blood samples for gas analyses were collected into 2 ml preheparinised syringes (BD A-Line, Becton, Dickinson & Company, New Jersey, USA) before induction of general anaesthesia (baseline), and at 2, 10 and 30 minutes after induction of general anaesthesia. The syringes were sealed immediately and the samples were analysed for blood gases within five minutes. Oxygen saturation (SaO_2_), oxygen concentration (PaO_2_), carbon dioxide tension (PaCO_2_), hydrogen ion concentration negative logarithm (pH) and bicarbonate ion ([HCO_3_^−^]) concentration were measured using a precalibrated blood gas analyser (Rapidlab 348 pH/Blood Gas and Electrolyte Analyser, Siemens Medical Solutions Diagnostics, Midrand, South Africa).

### Statistical analysis

Data were analysed using the R Statistical Software, V.2.7.2 (The R Foundation for Statistical Computing, Vienna, Austria). All data were assumed to be non-parametric because of the small sample size and are expressed as median (range).

Data on alfaxalone dose for induction, alfaxalone MIR, scores (quality of induction and recovery from anaesthesia), and times to extubation, standing and voluntary motion were tested for statistically significant differences among treatments using the Friedman rank sum test. If statistically significant differences were observed, a posthoc analysis (pair-wise Wilcoxon rank sum test with a Bonferroni adjustment for multiple testing) was conducted.

Repeatedly measured variables (HR, SAP, DAP, MAP, body temperature, respiratory rate, and blood-gas analyses data) were tested for statistically significant differences among and within treatments using the repeated measures analysis of variance by ranks followed by posthoc analysis (Tukey test).

A value of P<0.05 was considered to be significant.

## Results

The doses of alfaxalone required for induction and maintenance of general anaesthesia during treatment with midazolam in goats are summarised in Table 2. There were no statistically significant differences among the three treatments in the dose of alfaxalone required for induction of general anaesthesia. No incremental boli of alfaxalone were required to complete induction of general anaesthesia, over and above the initial bolus dose of 2 mg/kg except during LMID treatment. Induction of general anaesthesia was excitement-free and associated with easy intubation; with a perfect score of 2 (2–2) observed for all treatments.

**TABLE 2:** Observations [median (range)] regarding general anaesthestic induction dose and minimum infusion rate (MIR) of alfaxalone required to prevent movement of extremities in response to noxious stimulation during intravenous administration of midazolam: 0.1 mg/kg bolus followed by constant rate infusion (CRI) at 0.1 mg/kg/hour (LMID treatment), 0.3 mg/kg bolus followed by CRI at 0.3 mg/kg/hour (MMID treatment), or 0.9 mg/kg bolus followed by CRI at 0.9 mg/kg/hour (HMID treatment) in goats

Treatment	Alfaxalone induction dose (mg/kg)	Alfaxalone MIR (mg/kg/hour)	MIR determination time (minutes)
LMID	2.3 (2.0–2.5)	6.7 (6.7–8.6)	90 (60–83)
MMID	2.0 (2.0–2.0)	6.7 (4.8–6.7)	90 (90–120)
HMID	2.0 (2.0–2.0)	2.9 (1.0–4.8)*	150 (120–150)†

*Statistically significantly different (P<0.05) from LMID treatment

†Statistically significantly different (P<0.05) from both LMID and MMID treatments

**FIG 1:**
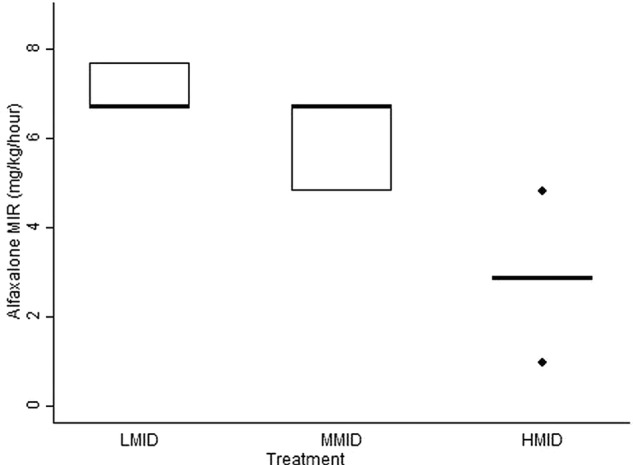
Box plot illustrating the minimum infusion rates of alfaxalone required to prevent movement of extremities in response to noxious stimulation during its co-administration with midazolam: 0.1 mg/kg bolus followed by constant rate infusion (CRI) at 0.1 mg/kg/hour (LMID treatment), 0.3 mg/kg bolus followed by CRI at 0.3 mg/kg/hour (MMID treatment), or 0.9 mg/kg bolus followed by CRI at 0.9 mg/kg/hour (HMID treatment) in goats

The median alfaxalone MIR of 2.9 (1.0–4.8) mg/kg/hour observed during HMID treatment was significantly higher than during LMID treatment. The relationship between alfaxalone MIR and the lidocaine treatments is illustrated in Fig 1. The median time to determination of alfaxalone MIR was similarly 90 minutes for LMID and MMID treatments, but increased significantly, to 150 minutes for HMID treatment.

A few significant differences within treatments were observed regarding the physiological parameters measured in the present study (Table 3). Most notably, at two minutes after the induction of general anaesthesia, SaO_2_ decreased significantly in comparison to baseline value within all three midazolam treatments. Arterial oxygen partial pressure (PaO_2_) was also significantly lower in comparison to baseline within all treatments at two minutes after induction of general anaesthesia. The hypoxaemia observed following the induction doses responded immediately to oxygen supplementation that was initiated soon after the two minutes parameter values had been recorded. Arterial carbon dioxide partial pressure also tended to increase gradually during the anaesthetic period and was significantly higher (but not clinically relevant) than baseline values towards the end of the anaesthestic period (around the time of alfaxalone MIR determination) within all treatments. A few clinically irrelevant significant differences from the baseline within treatments regarding HR, body temperature and pH were observed.

**TABLE 3:** Physiological parameters [median (range)] observed in goats in a study where the minimum infusion rate of alfaxalone during its co-administration with midazolam: 0.1 mg/kg bolus followed by constant rate infusion (CRI) at 0.1 mg/kg/hour (LMID treatment), 0.3 mg/kg bolus followed by CRI at 0.3 mg/kg/hour (MMID treatment), or 0.9 mg/kg bolus followed by CRI at 0.9 mg/kg/hour (HMID treatment) was determined

		Time of General Anaesthesia					
		Baseline	2 minutes	10 minutes	30 minutes	t-MIRα	t-MIRβ
Variable	Treatment	(Breathing room air)		(Oxygen Supplementation)			
Heart rate (beats/minute)	LMID	68 (59–96)	95 (84–104)	102 (85–113)*	104 (86–118)*	112 (86–125)*	123 (83–138)*
MMID	65 (60–73)	87 (82–95)	83 (78–94)	90 (82–105)	104 (82–125)*	116 (81–146)*	
HMID	70 (51–74)	83 (77–138)	81 (75–89)	86 (80–105)	104 (75–133)*	109 (96–136)*	
SAP (mm Hg)	LMID	105 (86–133)	96 (69–116)	104 (89–134)	108 (104–124)	108 (104–118)	107 (90–124)
	MMID	106 (93–125)	73 (63–119)	85 (75–137)	106 (85–123)	107 (78–130)	107 (83–139)
	HMID	105 (94–130)	69 (59–99)	80 (57–107)	95 (72–120)	111 (90–126)	120 (100–135)
DAP (mm Hg)	LMID	84 (55–91)	80 (60–111)	93 (71–109)	92 (86–106)	93 (86–104)	91 (78–111)
	MMID	84 (69–104)	51 (30–105)	67 (55–115)	88 (64–103)	89 (58–111)	92 (64–120)
	HMID	85 (65–100)	48 (25–69)	62 (44–87)	73 (53–95)	95 (75–104)	101 (86–109)
MAP (mm Hg)	LMID	96 (77–103)	86 (64–119)	97 (81–115)	99 (94–114)	98 (94–109)	97 (83–116)
	MMID	93 (81–112)	58 (41–102)	75 (70–124)	83 (61–106)	101 (81–112)	107 (91–119)
	HMID	92 (80–110)	57 (39–82)*	69 (54–95)*	93 (77–104)	98 (80–105)	95 (79–114)
Body temperature (°C)	LMID	38.9 (37.8–39.5)	38.5 (37.7–38.9)	38.2 (37.3–38.6)	38.0 (37.4–38.3)	37.8 (37.4–38.3)*	37.5 (37.3–38.2)*
	MMID	38.7 (38.3–39.1)	38.2 (37.6–38.9)	38.1 (37.3–38.7)	37.8 (37.0–38.3)	37.6 (36.8–38.0)*	37.2 (36.5–37.6)*
	HMID	39.0 (38.2–39.3)	38.7 (37.9–39.4)	38.4 (37.7–38.9)	38.0 (37.5–38.3)	37.5 (37.1–38.0)*	37.4 (36.8–37.9)*
Respiratory rate (breaths/minute)	LMID	26 (20–32)	20 (16–28)	16 (11–33)	18 (11–36)	19 (11–35)	17 (14–33)
	MMID	26 (20–32)	26 (9–38)	24 (11–39)	22 (16–40)	23 (14–34)	23 (13–38)
	HMID	28 (24–32)	21 (15–31)	25 (7–36)	30 (10–42)	25 (12–31)	18 (10–26)
F_I_O_2_	LMID	0.21 (0.21–0.21)	0.21 (0.21–0.21)	0.91 (0.69–0.95)	0.95 (0.9–0.97)		
	MMID	0.21 (0.21–0.21)	0.21 (0.21–0.21)	0.78 (0.57–0.91)	0.91 (0.86–0.95)		
	HMID	0.21 (0.21–0.21)	0.21 (0.21–0.21)	0.87 (0.77–0.96)	0.95 (0.93–0.97)		
S_a_O_2_ (%)	LMID	96.0 (94.4–96.7)	87.4 (80.9–92.8)*	99.6 (99.3–99.8)	99.7 (99.3–99.8)		
	MMID	96.1 (95.2–96.9)	85.0 (65.8–92.6)*	99.67 (99.3–99.8)	99.6 (99.2–99.8)		
	HMID	96.1 (95.2–96.7)	78.5 (62.9–94.3)*	99.6 (99.2–99.7)	99.6 (99.3–99.8)		
P_a_O_2_ (mm Hg)	LMID	73 (67–81)	51 (43–59)*	261 (118–394)*	285 (114–439)*		
	MMID	76 (70–84)	49 (37–71)*	265 (147–349)*	241 (187–323)*		
	HMID	75 (71–77)	44 (32–63)*	259 (182–326)*	272 (198–361)*		
P_a_CO_2_ (mm Hg)	LMID	37 (34–39)	42 (37–44)	46 (41–51)*	46 (42–55)*		
	MMID	36 (33–39)	45 (42–49)	50 (45–57)*	51 (46–57)*		
	HMID	37 (35–39)	45 (37–48)	49 (53–57)*	51 (47–57)*		
pH_a_	LMID	7.48 (7.46–7.54)	7.43 (7.37–7.42)	7.40 (7.38–7.49)*	7.39 (7.34–7.45)*		
	MMID	7.48 (7.46–7.57)	7.42 (7.38–7.44)*	7.38 (7.34–7.41)*	7.38 (7.36–7.42)*		
	HMID	7.48 (7.47–7.60)	7.41 (7.38–7.52)*	7.37 (7.33–7.42)*	7.37 (7.31–7.41)*		
[HCO_3_^−^] (mmol/l)	LMID	27.3 (24.8–30.4)	26.6 (24.5–30.0)	28.0 (24.8–30.0)	27.8 (23.9–30.7)		
	MMID	26.2 (24.1–29.2)	28.1 (24.2–30.3)	28.6 (26.9–31.5)	30.1 (28.2–32.9)		
	HMID	27.6 (25.3–35.9)	28.5 (25.6–30.3)	27.8 (25.3–30.0)	29.0 (27.0–31.7)		

t-MIR_α_, time at which alfaxalone infusion last abolished purposeful movement (lowest effective alfaxalone CRI rate)

t-MIR_β_, time at which purposeful movement was observed and anaesthesia discontinued

There were no statistically significant differences observed among treatment observations at same time points

*Statistically significantly different (P<0.05) from baseline observation within treatment

[HCO_3_^−^], arterial bicarbonate ion concentration; DAP, diastolic arterial pressure; F_I_O_2,_ fractional inspired oxygen; f_R_, respiratory frequency; MAP, mean arterial pressure; MIR, minimum infusion rate; P_a_CO_2_, partial pressure of carbon dioxide in arterial blood; P_a_O_2_, partial pressure of oxygen in arterial blood; pHa, arterial hydrogen ion concentration negative logarithm; S_a_O_2_, saturation of haemoglobin with oxygen in arterial blood; SAP, systolic arterial pressure

At no point after two minutes of general anaesthesia did any goat require rescue assisted ventilation for a high end-tidal carbon dioxide tension or low SpO_2_. The median body temperature of the goats was maintained between 37.2°C and 39.0°C, but the body temperature decreased below 37.0°C towards the end of the anaesthetic procedure during MMID and HMID treatment in a few goats. The only other adverse effect was rumen tympany, which was present in all goats by the end of the anaesthetic procedure.

Recovery from anaesthesia was excitement-free following all the treatments (Table 4). Treatment HMID caused statistically significantly longer times to standing and voluntary motion in comparison to LMID.

**TABLE 4:** Quality of recovery from anaesthesia [median (range)] observed in goats in a study where the minimum infusion rate of alfaxalone during its co-administration with midazolam: 0.1 mg/kg bolus followed by constant rate infusion (CRI) at 0.1 mg/kg/hour (LMID treatment), 0.3 mg/kg bolus followed by CRI at 0.3 mg/kg/hour (MMID treatment), or 0.9 mg/kg bolus followed by CRI at 0.9 mg/kg/hour (HMID treatment) was determined

	Time to extubation (minutes)	Time to standing (minutes)	Time to voluntary motion (minutes)	Recovery score
LMID	9 (2–30)	61 (43–77)	61 (43–77)	3 (3–3)
MMID	12 (8–20)	79 (50–180)	79 (50–180)	3 (3–3)
HMID	24 (10–30)	135 (75–160)*	135 (75–160)*	3 (3–3)

*Statistically significantly different (P<0.05) from LMID treatment

## Discussion

The present study demonstrates that midazolam CRI has an adjunctive role in alfaxalone-based TIVA in goats, but caution must be exercised when using high doses of midazolam as hypoxaemia during anaesthestic induction and prolonged recovery times are potential adverse effects.

Midazolam CRI reduced the alfaxalone dose for induction of general anaesthesia in a dose-dependent manner in the present study. It is most likely that the alfaxalone dose required for induction would have been less than the fixed initial (possible minimum) dose of 2 mg/kg used in the present study during HMID treatment had alfaxalone been titrated to effect from a lower dose. Midazolam has previously been reported to be a potent sedative and hypnotic drug (Stegmann and Bester 2001) that can be administered during the preanaesthetic period to reduce the dose of alfaxalone (Dzikiti and others 2014) or propofol (Dzikiti and others 2009) required for induction of general anaesthesia in goats.

The observation of excitement-free inductions that facilitated easy intubation, irrespective of the treatments administered in the present study confirms outcomes of a recent study, in which a similar conclusion was reached even in sedated and un-sedated goats during induction of general anaesthesia with alfaxalone (Dzikiti and others 2014).

The alfaxalone MIRs observed in the present study following administration of LMID, MMID and HMID treatments in goats represent reductions of about 30, 30 and 70 per cent, respectively, in comparison to a previously reported alfaxalone MIR in unsedated goats of 9.6 mg/kg/hour (Ndawana and others 2014). This demonstrates an important and dose-dependent effect of midazolam on the dose of alfaxalone required for maintenance of general anaesthesia. In a previous study, midazolam infusions administered at similar doses, reduced isoflurane requirements for maintenance of general anaesthesia in goats by 17 (13–19) per cent, 35 (30–41) per cent and 55 (49–56) per cent following LMID, MMD and HMID respectively (Dzikiti and others 2011). This comparison shows that midazolam produces marked reductions in drug requirements for general anaesthesia in both inhalation and intravenous anaesthesia in goats, with an evidently more pronounced reduction in alfaxalone than isoflurane requirements. The substantive reduction in alfaxalone requirements demonstrates that midazolam can be used as an adjunct to alfaxalone-based TIVA in goats. Reducing the doses of drugs used for maintenance of general anaesthesia by combining different anaesthetic drugs (balanced anaesthesia), has the potential to obtund the occurrence of adverse dose-dependent cardiopulmonary effects associated with general anaesthetic drugs such as alfaxalone (Dundee and McMurray 1984, Hasley 1991, Mani and Morton 2010, Dzikiti 2013).

Physiological parameters assessed in the present study were minimally affected by co-administration of midazolam and alfaxalone for TIVA with the only period of concern being at two minutes of induction of general anaesthesia when cardiopulmonary depression, typified by hypotension (MAP<60 mm Hg) and hypoxaemia (SaO_2_<90 per cent) especially worse at higher doses of midazolam, was evident. It is mostly likely that the cardiopulmonary depression observed in the present study was a result of a combination of factors that include; (1) the high doses of midazolam, (2) the fixed initial induction bolus dose of alfaxalone at 2 mg/kg, which might be an excessive dose in combination with MMID and HMID treatments, (3) the rate at which both midazolam and alfaxalone were administered and (4) the delayed initiation of oxygen supplementation after induction. Both midazolam and alfaxalone have previously been associated with minimal cardiopulmonary depression in goats (Dzikiti and others 2010, Dzikiti and others 2011, Ndawana and others 2014). In previous studies, in which midazolam was co-administered with either propofol or isoflurane for induction and maintenance of general anaesthesia in goats, MAP and SpO_2_ were not adversely affected at any time during the experimental procedure (Dzikiti and others 2010, Dzikiti and others 2011). In a recent study, co-administration of midazolam and alfaxalone for induction of general anaesthesia in goats, minimally affected arterial blood pressure and tissue oxygenation (Ndawana and others 2014). The increased risk of development of hypoxaemia and hypotension observed in the present study justifies a need for cautious titration of alfaxalone to effect and quicker commencement of oxygen supplementation when high doses of midazolam are used as preanaesthetic medication for induction of general anaesthesia with alfaxalone in goats.

The quality of recovery from anaesthesia was good following all treatments, but HMID caused substantive prolongation of the recovery period. This observation is in contrast to outcomes of a previous study in which midazolam did not cause any substantive delays in recovery from isoflurane anaesthesia in goats (Dzikiti and others 2011). That some goats took longer than two hours to stand following termination of anaesthesia, suggests that the interaction of high doses of midazolam and alfaxalone causes significant prolongation of recovery from anaesthesia.

The results of the present study should be interpreted cautiously as they are deduced from a small sample size drawn from a single breed of goats and only basic physiological parameters were assessed, while pharmacokinetic analyses of the drugs involved were not performed.

In conclusion, midazolam CRI causes substantive and dose-dependent reduction of the dose of alfaxalone required for maintenance of general anaesthesia with minimal adverse effects on cardiopulmonary function in spontaneously-breathing goats. Vigilant titration of alfaxalone and early provision of supplementary oxygen is recommended during induction of general anaesthesia, especially if high doses of midazolam have been used for preanaesthetic medication, to minimise the risk of hypoxaemia. Recovery from alfaxalone TIVA may be delayed by high doses of midazolam.

## References

[R1] Andaluz A., Felez-ocana N., Santos L., Fresno L., Garcia F. (2012) The effects on cardio-respiratory and acid-base variables of the anaesthetic alfaxalone in a 2-hydroxypropyl-β-cyclodextrin (HPCD) formulation in sheep. Veterinary Journal 191, 389–392 10.1016/j.tvjl.2011.03.017 21543243

[R2] Dundee J. W., McMurray T. J. (1984) Clinical aspects of total intravenous anaesthesia: discussion paper. Journal of the Royal Society of Medicine 77, 669–672 6148418 10.1177/014107688407700811 PMC1440119

[R3] Dzikiti T. B. (2013) Intravenous anaesthesia in goats: a review. Journal of the South African Veterinary Association 84, E1–E8. dx.doi.org/10.4102/jsava.v84i1.499. Accessed May 26, 2014 23718851 10.4102/jsava.v84i1.499

[R4] Dzikiti T. B., Stegmann G. F., Dzikiti L. N., Hellebrekers L. J. (2010) Total intravenous anaesthesia (TIVA) with propofol-fentanyl and propofol-midazolam combinations in spontaneously breathing goats. Veterinary Anaesthesia and Analgesia 37, 519–525 21072973 10.1111/j.1467-2995.2010.00568.x

[R5] Dzikiti T. B., Stegmann G. F., Dzikiti L. N., Hellebrekers L. J. (2011) Effects of midazolam on isoflurane minimum alveolar concentration and cardiovascular function goats. Small Ruminant Research 97, 104–109 10.1136/vr.d999 21493442

[R6] Dzikiti T. B., Stegmann G. F., Hellebrekers L. J., Dzikiti L. N. (2009) Sedative and cardiopulmonary effects of acepromazine, midazolam, butorphanol, acepromazine-butorphanol and midazolam-butorphanol on propofol anaesthesia in goats. Journal of the South African Veterinary Association 80, 10–16 19653513 10.4102/jsava.v80i1.162

[R7] Dzikiti T. B., Zeiler G. E., Dzikiti L. N., Garcia E. R. (2014) The effects of midazolam and butorphanol, administered alone or combined, on the dose and quality of anaesthetic induction with alfaxalone in goats. Journal of the South African Veterinary Association 85, Art. #1047, 8 pages. dx.doi. org/10.4102/jsava.v85i1.1047. www.jsava.co.za/index.php/jsava/article/viewFile/1047/1403. Accessed September 19, 2014 10.4102/jsava.v85i1.1047 25686277

[R8] Farkas K. T., Tarnawa I., Berzsenyi P. (1989) Effects of centrally-acting muscle relaxants on spinal root potentials: a comparative study. Neurophysiology 28, 161–173 10.1016/0028-3908(89)90053-1 2716970

[R9] Ferré P. J., Pasloske K., Whittem T., Ranasinghe M., Li Q., Lefebvre H. P. (2006) Plasma pharmacokinetics of alfaxalone in dogs after an intravenous bolus of Alfaxan-CD RTU. Veterinary Anaesthesia and Analgesia 33, 229–236 16764587 10.1111/j.1467-2995.2005.00264.x

[R10] Ghurashi M. A. H., Seri H. I., Bakheit A. H., Ashwag E. A. M., Abakar J. A. (2009) Evaluation of ketamine/diazepam anaesthesia for performing surgery in desert goats under field conditions. Australian Journal of Basic and Applied Sciences 3, 455–459

[R11] Hall L. W., Chambers J. P. (1987) A clinical trial of propofol infusion anaesthesia in dogs. Journal of Small Animal Practice 28, 623–637

[R12] Hasley M. J. (1991) Occupational health and pollution from anaesthetics: a report of a seminar. Anaesthesia 46, 486–488 2048672 10.1111/j.1365-2044.1991.tb11692.x

[R13] Hendrickx J. F. A., Eger E. I. II, Sonner J. M., Schafer S. L. (2008) Is synergy the rule: a review of anaesthetic interactions producing hypnosis and immobility. Anesthesia and Analgesia 107, 494–506 18633028 10.1213/ane.0b013e31817b859e

[R14] Kaul H. L., Bharti N. (2002) Monitoring depth of anaesthesia. Indian Journal of Anaesthesia 46, 323–332

[R15] Klöppel H., Leece A. E. (2011) Comparison of ketamine and alfaxalone for induction and maintenance of anaesthesia in ponies undergoing castration. Veterinary Anaesthesia and Analgesia 38, 37–43 21214708 10.1111/j.1467-2995.2010.00584.x

[R16] Lemke K. A. (2007) Anticholinergics and sedatives. In Lumb and Jones's Veterinary Anaesthesia and Analgesia. 4th edn Eds Tranquilli W. J., Thurmon J. C., Grimm K. A. Blackwell Publishers pp 203–239

[R17] Mani V., Morton N. S. (2010) Overview of total intravenous anaesthesia in children. Pediatric Anesthesia 20, 211–222 19694975 10.1111/j.1460-9592.2009.03112.x

[R18] Mathis A., Pinelas R., Brodbelt D. C., Alibhai H. K. (2012) Comparison of quality of recovery from anaesthesia in cats induced with propofol or alfaxalone. Veterinary Anaesthesia and Analgesia 39, 282–290 22486806 10.1111/j.1467-2995.2011.00707.x

[R19] Ndawana P. S., Dzikiti B. T., Zeiler G. E., Dzikiti L. N. (2014) Determination of the Minimum Infusion Rate (MIR) of alfaxalone required to prevent purposeful movement of the extremities in response to a standardised noxious stimulus in goats. Veterinary Anaesthesia and Analgesia. doi:10.1111/vaa.12162. onlinelibrary.wiley.com/doi/10.1111/vaa.12162/pdf 10.1111/vaa.12162 24674097

[R20] Olkkola K. T., Ahonen J. (2008) Midazolam and other benzodiazepines. In Modern Anesthetics – Handbook of Experimental Pharmacology. Vol 182. Eds Schüttler J., Schwilden H. Springer Publishers pp 335–360 10.1007/978-3-540-74806-9_16 18175099

[R21] Quasha A. L., Eger E. I. II, Tinker J. H. (1980) Determination and applications of MAC. Anesthesiology 53, 315–334 6107067 10.1097/00000542-198010000-00008

[R22] Sear J. W., Phillips K. C., Andrews C. J. H., Prys-Roberts C. (1983) Dose-response relationships of infusions of althesin or methohexitone. Anesthesia 38, 931 10.1111/j.1365-2044.1983.tb12021.x 6638440

[R23] Stegmann G. F., Bester L. (2001) Sedative-hypnotic effects of midazolam in goats after intravenous and intramuscular administration. Veterinary Anaesthesia and Analgesia 28, 49–55 10.1046/j.1467-2987.2000.00034.x 28404003

[R24] Suarez M. A., Dzikiti B. T., Stegmann F. G., Hartman M. (2012) Comparison of alfaxalone and propofol administered as total intravenous anaesthesia for ovariohysterectomy in dogs. Veterinary Anaesthesia and Analgesia 39, 236–244 22405473 10.1111/j.1467-2995.2011.00700.x

[R25] Taira Y., Nakakimura K., Matsumoto M., Sakabe T. (2000) Spinal and supraspinal midazolam potentiates antinociceptive effects of isoflurane. British Journal of Anaesthesia 85, 881–886 11732524 10.1093/bja/85.6.881

